# A Co-Evolution Model for Dynamic Social Network and Behavior

**DOI:** 10.4236/ojs.2014.49072

**Published:** 2014-10

**Authors:** Liping Tong, David Shoham, Richard S. Cooper

**Affiliations:** Department of Public Health Sciences, Loyola University Medical School, Maywood, USA

**Keywords:** Social Network, Social Behavior, Co-Evolution, Markov Chain, Stationary Distribution

## Abstract

Individual behaviors, such as drinking, smoking, screen time, and physical activity, can be strongly influenced by the behavior of friends. At the same time, the choice of friends can be influenced by shared behavioral preferences. The actor-based stochastic models (ABSM) are developed to study the interdependence of social networks and behavior. These methods are efficient and useful for analysis of discrete behaviors, such as drinking and smoking; however, since the behavior evolution function is in an exponential format, the ABSM can generate inconsistent and unrealistic results when the behavior variable is continuous or has a large range, such as hours of television watched or body mass index. To more realistically model continuous behavior variables, we propose a co-evolution process based on a linear model which is consistent over time and has an intuitive interpretation. In the simulation study, we applied the expectation maximization (EM) and Markov chain Monte Carlo (MCMC) algorithms to find the maximum likelihood estimate (MLE) of parameter values. Additionally, we show that our assumptions are reasonable using data from the National Longitudinal Study of Adolescent Health (Add Health).

## 1. Introduction

Numerous studies have examined the role friends play in influencing behavior. Researchers have made extensive use of data from the Framingham Heart Study-Network Study (FHS-Net) [1]-[3], the National Longitudinal Study of Adolescent Health (Add Health) [4] [5], and other datasets [6]-[9] to examine whether health behaviors such as smoking and becoming obese can spread between friends. However, the validity of analyses based on observational studies has been called into question by several authors [10] [11]. The main concern is the impossibility of identification of peer influence from peer selection using regression-based approaches [11].

In response to these concerns, the actor-based stochastic model (ABSM) was proposed by [12] [13]. This model employs Markov chain simulation and method-of-moments (MOM) to adjust estimates of peer influence and peer selection parameters using longitudinal data. The underlying model is a random utility function, where the utilities are not observed. This type of model is the most appropriate for scenarios where an actor must make a single choice from a given set of choices [14], although several researchers have applied the ABSM model to continuous behaviors [7] [8].

In ABSM, a continuous time finite-state-space Markov process was used to model the dynamic relationship between social network and behaviors. Three steps describe this process. The first step determines when the chance for the next change will occur. Let $\lambda_{i}^{n}$ be the rate of change for actor *i*’s network and $\lambda_{i}^{b}$ be the rate of change for actor *i*’s behavior. Then the waiting time for the next chance of change is exponentially distributed with parameter $\lambda =\sum_{i}\left(\lambda_{i}^{n}+\lambda_{i}^{b}\right)$. Note that the chance of change does not necessarily results in successful change. The second step defines which actor has the opportunity to make a change (either a network change or a behavior change). The probability of a network change taken by a particular actor *i* is given by $\lambda_{i}^{n}/\lambda$ and the probability that this is a behavior change taken by actor *i* is $\lambda_{i}^{b}/\lambda$. At the third step there is an opportunity to make a change in network or behavior. If actor *i* is making a network change, there are *n* possible outcomes, where *n* is the number of actors in the network. This condition holds because for network changes, at most one tie difference from the current network is allowed; no network change is also allowed. Say, *y* is the current network. The next network *y*′ must be either equal to *y* or deviate from *y* exactly one element in row *i*. To simplify notation, for adjacent matrix *y* and indicators *i* = 1, 2, ···, *n* and *j* = 1, 2, ···, *n*, we define a mapping function *c*(*y*, *i*, *j*) that maps *y* to a new matrix *y*′ whose (*s*, *t*)th element $y_{st}^{\prime }$ equals the (*s*, *t*)th element of *y*, which is *y_st_*, when *s* ≠ *i* or *t* ≠ *j*. If *s* = *i* and *t* = *j*, there are two situations. If *j* = *i*, then $y_{ij}^{\prime }=y_{ij}$. If *j* ≠ *i*, then $y_{ij}^{\prime }=1-y_{ij}$. For actor *i*, let *u_ik_* be the *k* th effect for network evolution, which is a function of network variable *y* or behavior variable *z*, or both. Therefore, we can write *u_ik_* = *u_ik_*(*y*, *z*) to emphasize this relationship. Then the network objective function of actor *i* is $f_{i}^{n}(y,z)=\sum_{k\in K}\beta_{k}^{n}u_{ik}(y,z)$ where $\beta_{k}^{n}$ are parameters that are either given (in a simulation) or estimated from data (in a analysis), and 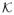 is a set of the effects of interest. The probability that actor *i* will make a network change and have a new network value *y*′ = *c*(*y*, *i*, *j*) is

(1) $$P\left(Y_{t}=y^{\prime }|Y_{t-1}=y,Z_{t-1}=z\right)=\frac{\exp\left(f_{i}^{n}\left(y^{\prime },z\right)\right)}{\sum_{t=1}^{n}\exp\left(f_{i}^{n}\left(c\left(y,i,t\right),z\right)\right)}.$$

If actor *i* is going to make a behavior change, there are 3 possible outcomes: increase 1 unit, stay the same, or decrease 1 unit. Similarly, for *i* = 1, 2, ···, *n* and *j* = 1, 2, 3, define a mapping function *d*(*z*, *i*, *j*) that maps vector *z* to a new vector *z*′ whose *s*th element $z_{s}^{\prime }$ equals to the *s* th element of *z*, which is *z_s_*, when *s* ≠ *i*. If *s* = *i*, then $z_{i}^{\prime }=z_{i}+j-2$ for *j* = 1, 2, 3. For actor *i*, let *v_ik_*(*y*, *z*) be the *k* th effect for behavior evolution. Then the behavior objective function of actor *i* is $f_{i}^{b}(y,z)=\sum_{k\in K}\beta_{k}^{b}v_{ik}\left(y,z\right)$. The probability that actor *i* will make a behavior change and have a new behavior value *z*′ = *d*(*z*, *i*, *j*) is

(2) $$P\left(Z_{t}=z^{\prime }|Y_{t-1}=y,Z_{t-1}=z\right)=\frac{\exp\left(f_{i}^{b}\left(y,z^{\prime }\right)\right)}{\sum_{t=1}^{3}\exp\left(f_{i}^{b}\left(y,d\left(z,i,t\right)\right)\right)}.$$

In summary, the probability to change to a new set of value (*y*′, *z*′) in the next step is

(3) $$P\left(Y_{t}-y^{\prime },Z_{t}=z^{\prime }|Y_{t-1}=y,Z_{t-1}=z\right)=\left\{ \begin{array}{ll} \frac{\lambda_{i}^{n}}{\lambda }\times \frac{\exp\left(f_{i}^{n}\left(y^{\prime },z\right)\right)}{\sum_{t=1}^{n}\exp\left(f_{i}^{b}\left(c\left(y,i,t\right),z\right)\right)} & \text{if}\;z^{\prime }=z\;\text{and}\;y^{\prime }=c(y,i,j),1\leq i\leq n\;\text{and}\;1\leq j\leq n,\\ \frac{\lambda_{i}^{b}}{\lambda }\times \frac{\exp\left(f_{i}^{b}\left(y^{\prime },z\right)\right)}{\sum_{t=1}^{3}\exp\left(f_{i}^{b}\left(y,d\left(z,i,t\right)\right)\right)} & \text{if}\;y^{\prime }=y\;\text{and}\;z^{\prime }=d(z,i,j),1\leq i\leq n\;\text{and}\;1\leq j\leq 3,\\ 0 & \text{otherwise}. \end{array}\right.$$

To use ABSM, the behavior variable must be bounded and discretized. For continuous behavior variables, such as body mass index (BMI), time spent watching television, etc., the process of discretizing can be arbitrary and tricky. In Section 3 (Results), we show that the effect of average BMI similarity can be very different for integer and categorical BMI.

Based on the above considerations, we were motivated to develop a linear-based behavior evolution model. In our model, the network evolution is similar to ABSM. However, the behavior evolution is defined by a continuous Markov process, which is completely different from [12] [13]. To simplify computation, we consider only a real network change as an “event” (instead of the opportunity of change). In addition, for behavior evolution, we assume normal residuals for values of change.

## 2. Methods

### 2.1. Complete and Observed Data

For illustration purpose, consider two waves of data that are collected at time 0 and *T*. The complete data during time period (0, *T*) include number of events *k*, time of events *t*_0_ = 0, *t*_1_, ···, *t_k_*, *y*^0^, *y*^1^, ···, *y^k^*, *y*^T^ = *y^k^* (or write as *y*^0^, (*i*_(1)_, *j*_(1)_), ···, (*i*_(_*_k_*_)_, *j*_(_*_k_*_)_), where (*i*_(_*_s_*_)_, *j*_(_*_s_*_)_), 1 ≤ *i*_(_*_s_*_)_ ≠ *j*_(_*_s_*_)_ ≤ *n*, is the network edge changing at time *t_s_*, *s* = 1, ···, *k*), and behavior variable *z*^0^, *z*^1^, ···, *z^k^*, *z*^T^ = *z^k^*^+1^. The observed data include network variables *y*^0^, *y*^T^ and behavior variables *z*^0^, *z*^T^. All the other variables occur between observations, and thus are considered missing in the complete data set. The joint evolution of network and behavior is shown in the following flow chart:


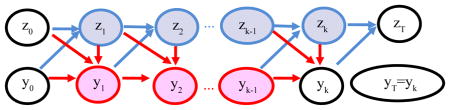


Here the observed data are represented in black ovals, missing behavior data in blue ovals, and missing network data in red ovals. The network evolution process is represented by red arrows and behavior variable by blue arrows.

### 2.2. Occurrence of Events

The number of events *k* during time period (0, *T*) follows a Poisson distribution with rate *λT*. Conditional on *k*, the event times *t*_1_, ···, *t_k_* has the joint probability density function

(4) $$f\left(t_{1},\cdots ,t_{k}|k\right)=\left\{ \begin{array}{ll} k!/T^{k} & \text{if}\;0\leq t_{1}\leq \cdots \leq t_{k}\leq T,\\ 0 & \text{otherwise}. \end{array}\right.$$

For now, we assume the chance of making a network change is the same for each actor. This assumption can be extended to be actor-specific if the data are informative enough.

### 2.3. Network Evolution

Let *u*(*y*, *z*) be an arbitrary vector of statistics of the graph and behavior, *θ* be the vector of coefficients, and *ψ*(*θ*) be the normalizing factor. Define $\delta_{ij}\left(y,z\right)=u\left(y_{ij}^{+},z\right)-u\left(y_{ij}^{-},z\right)$, where $y_{ij}^{+}$ is the same as *y* except that the edge (*i*, *j*), ${\left(y_{ij}^{+}\right)}_{ij}=1$. Likewise, ${\left(y_{ij}^{-}\right)}_{ij}=0$. If the current network is *y* and the behavior immediately before the next event is *z*, the probability to change edge (*i*, *j*) at next event is

(5) $$P\left\{\text{change}(i,j)\right\}=\frac{1}{n}\cdot \frac{\exp\left\{\left(2y_{ij}-1\right)\theta^{T}\delta_{ij}\left(y,z\right)\right\}}{\sum_{j^{\prime }\neq i}\exp\left\{\left(2y_{{ij}^{\prime }}-1\right)\theta^{T}\delta_{{ij}^{\prime }}\left(y,z\right)\right\}}.$$

### 2.4. Behavior Evolution

Define Δ*Z*(*t*_1_, *t*_2_)=*Z*^t_2_^ − *Z*^*t*_1_^, the vector of behavior variable changes from time *t*_1_ to *t*_2_. For any time *t* ∈ (*t_u_*, *t_u_*_+1_), we propose the following co-evolution model for behavior variables

(6) $$\Delta Z\left(t_{u},t\right)=\alpha W^{u}\Delta Z\left(t_{u},t\right)+\left(t-t_{u}\right)X\gamma +\varepsilon^{u}$$

where *W^u^* = ()*_n_*_×_*_n_* is a matrix of functions of *y*^*t*_*u*_^, *X* = ()*_n_*_×_*_p_* is the matrix of *p* covariates, *γ* = (*γ*_1_, ···, *γ_p_*)^T^ is the vector of coefficients for general trend for BMI, $\varepsilon^{u}={\left(\varepsilon_{1}^{u},\cdots ,\varepsilon_{n}^{u}\right)}^{T}$ are all independent from each other or any other random variable, and *ε^u^* follows a multi-dimensional normal distribution with mean zero and variance matrix (*t* − *t_u_*)*σ*^2^*I_n_*. Note that *W^u^* represents individuals’ friendship network variables. This is saying that the change of individuals’ behavior is a function of friends’ behavior change. The parameter *α* measures how strong this relationship is. In the next subsection we give an example choice of *W* and explain this function more intuitively.

Note that when *t* = *t_u_*, the variance of *ε^u^* is zero and therefore there is no change at all; when *t* increases, the variance of *ε^u^* increases, as one would expect. Equation (6) can also be written as

(7) $$(I-\Delta \alpha W^{u})Z\left(t_{u},t\right)=\left(t-t_{u}\right)X\gamma +\varepsilon^{u}.$$

Since behavior variables are accumulated over time, we would expect that when modeling behaviors, the distribution of change from time *t_u_* to *t_u_*_+1_ is consistent with a two-step process: first from *t_u_* to *t*, then from *t* to *t_u_*_+1_. In our model, this condition is naturally satisfied because

$$(I-\alpha W_{u})\Delta Z\left(t_{u},t_{u+1}\right)=\left(I-\alpha W_{u}\right)\left[\left(Z^{t_{u+1}}-Z^{t}\right)+\left(Z^{t}-Z^{t_{u}}\right)\right]=\left(t_{u+1}-t_{u}\right)X\gamma +\left(\varepsilon^{u}+\varepsilon^{t}\right)$$

where *ε^u^* and *ε^t^* are independent and both follow multi-dimensional normal distributions with mean zero and variances (*t* − *t_u_*)*σ*^2^*I_n_* and (*t_u_*_+1_ − *t*)*σ*^2^*I_n_* respectively, which indicates that *ε^u^* + *ε^t^* follows a normal distribution with mean zero and variance (*t_u_*_+ 1_ − *t_u_*)*σ*^2^*I_n_*. This is exactly what we expect. Note that in ABSM [7] [8], this condition is usually not satisfied for continuous behavior variables.

### 2.5. An Example Choice of *W*

As an example, assume that the *i* th individual’s BMI change during time (*t*_1_, *t*_2_), where 0 ≤ *t*_1_ < *t*_2_ ≤ *T*, is a linear function of the average change of BMI of his/her friends. That is,

$$\Delta Z_{i}=\alpha \sum_{j}y_{i,j}\Delta Z_{j}/\sum_{j}y_{ij}+\varepsilon_{i}$$

where *ε_i_* is independent of any other random variable and follows a normal distribution with mean zero and variance (*t*_2_ − *t*_1_)*σ*^2^. When written in matrix format:

ΔZ=αWΔZ+ε

where *W* = (diag(*y*_1+_, ···, *y_n_*_+_))^−1^ *y*. That is, (*I_n_* − *αW*) Δ*Z* = *ε*. We can then assume that the corrected behavior variables (*I_n_* − *αW*) Δ*Z* follow a multi-dimensional normal distribution with mean 0 and variance matrix (*t*_2_ − *t*_1_)*σ*^2^*I_n_*. A network effect exists if *α* ≠ 0.

### 2.6. Complete Data Log-Likelihood Function

Exponential random graph models (ERGMs) are commonly employed to test whether the presence of network ties (edges) differs from what would be expected in a random graph, given some set of network statistics [15]. In the ERGM, the parameters are *η* = (*λ*, *θ*, *γ*, *α*, *σ*) with dimension = 3 + *p* +*q*, where *p* is the number of covariates and *q* is the number of network statistics in the ERGM. The complete data log-likelihood function is

(8) $$\begin{array}{l} l(\eta )=-\lambda T+k\text{log}(\lambda )+\sum_{u=1}^{k}\left(2y_{i_{\left(u\right)},j_{\left(u\right)}}^{u-1}-1\right)\theta^{T}\delta_{i_{\left(u\right)},j_{\left(u\right)}}\left(y^{u-1},z^{u}\right)-\sum_{u=1}^{k}\log\left(\sum_{j^{\prime }\neq i_{\left(u\right)}}\exp\left(\left(2y_{i_{\left(u\right)},j^{\prime }}^{u-1}-1\right)\theta^{T}\delta_{i_{\left(u\right)},j^{\prime }}\left(y^{u-1},z^{u}\right)\right)\right)\\ -\frac{n}{2}\sum_{u=1}^{k+1}\log\sigma^{2}-\frac{n}{2}\sum_{u=1}^{k+1}\log\left(t_{u}-t_{u-1}\right)+\sum_{u=1}^{k+1}\log\left|I-\alpha W^{u-1}\right|-\sum_{u=1}^{k+1}\frac{{\left(\left(I-\alpha W^{u-1}\right)z^{u}-\mu_{u}\right)}^{T}\left(\left(I-\alpha W^{u-1}\right)z^{u}-\mu_{u}\right)}{2\left(t_{u}-t_{u-1}\right)\sigma^{2}} \end{array}$$

where *μ_u_* = (*I*−*αW^u^*^−1^)*z^u^*^−1^ + (*t_u_*−*t_u_*_−1_)*X_γ_*.

### 2.7. EM Algorithm to Find MLE of Parameters

Parameter *λ* can be estimated directly: *λ̂*_MLE_ = *E* (*k*|*y*_0_, *z*_0_, *y_T_*, *z_T_*)/*T*. The EM algorithm to estimate the other parameters can be described as follows: 1) Start from initial values *η*_0_; 2) E-step: calculate *h*(*η*) = *E*_*η*_0__ (*l* (*η*)(*y*^0^, *z*^0^, *y*^T^, *z*^T^)); 3) M-step: maximize *h*(*η*) over the parameter space to update *η*; 4) With the new value of *η*, repeat the E- and M-steps. Since the E-step cannot be calculated directly, we use Markov Chain Monte Carlo to simulate hidden variables *R* times. We evaluate the complete data log-likelihood function using simulated samples and obtain *l*^1^(*η*),⋯,*l^R^* (*η*). Then $h(\eta )\approx \frac{1}{R}\sum_{u=1}^{R}l^{u}(\eta )$ For the M-step, the MLE of parameters (*λ, γ, σ*) can be written as functions of the MLE of parameters (*θ, α*). Then *h*(*η*) becomes a smoothed function of (*θ, α*), which can be maximized using computational methods. Specifically,

$$\begin{array}{l} {\hat{\gamma }}_{\text{MLE}}={\left(R\left(T-t_{0}\right)X^{T}X\right)}^{-1}\sum_{v=1}^{R}\sum_{u=1}^{k+1}X^{T}\left(I-\alpha W^{u-1}\right)\Delta z\left(t_{u-1},t_{u}\right),\\ {\hat{\sigma }}_{\text{MLE}}^{2}=\frac{1}{n\left(k+1\right)R}\sum_{v=1}^{R}\sum_{u=1}^{k+1}\frac{{\left(A^{u}-\left(t_{u}-t_{u-1}\right)X{\hat{\gamma }}_{\text{MLE}}\right)}^{T}\left(A^{u}-\left(t_{u}-t_{u-1}\right)X{\hat{\gamma }}_{\text{MLE}}\right)}{t_{u}-t_{u-1}}, \end{array}$$

where *A^u^* = (*I*−*αW^u^*^−1^)Δ*z*(*t_u_*_−1_,*t_u_*),

$$h(\eta )=\text{constant}+\sum_{v=1}^{R}\sum_{u=1}^{k}\left(2y_{i_{\left(u\right)},j_{\left(u\right)}}^{u-1}-1\right)\theta^{T}\delta_{i_{\left(u\right)},j_{\left(u\right)}}\left(y^{u-1},z^{u}\right)-\sum_{v=1}^{R}\sum_{u=1}^{k}\log\left(\sum_{j^{\prime }\neq i_{\left(u\right)}}\exp\left(\left(2y_{i_{\left(u\right)}j^{\prime }}^{u-1}-1\right)\theta^{T}\delta_{i_{\left(u\right)}j^{\prime }}\left(y^{u-1},z^{u}\right)\right)\right)+\frac{Rn(k+1)}{2}\log{\hat{\sigma }}_{\text{MLE}}^{2}+\sum_{v=1}^{R}\sum_{u=1}^{k+1}\log\left|I-\alpha W^{u-1}\right|.$$

### 2.8. Normal Distribution to Simulate Behavior Variable *Z^u^*

In the general multi-dimensional situation, assume that *X*_1_ ~ *N* (0,Σ_1_), *X*_2_ ~ *N*(0,Σ_2_), *X*_1_ and *X*_2_ are independent. Then *X*_1_ + *X*_2_ ~ *N* (0,Σ_1_ + Σ_2_). The distribution of *X*_1_ conditional on *X* = *X*_1_ + *X*_2_ is normal with mean ${\left(I_{n}+\sum_{2}\sum_{1}^{-1}\right)}^{-1}x$ and variance ${\left(\sum_{1}^{-1}+\sum_{2}^{-1}\right)}^{-1}$. In our situation, for *u* = 1,⋯, *k*−1, we have

$$\sum_{1}=\left(t_{u}-t_{u-1}\right)\sigma^{2}{\left(\left(I-\alpha W^{u-1}\right){\left(I-\alpha W^{u-1}\right)}^{T}\right)}^{-1}$$

and

$$\sum_{2}=\sum_{h=u+1}^{k}\left(t_{h}-t_{h-1}\right)\sigma^{2}{\left(\left(I-\alpha W^{h-1}\right){\left(I-\alpha W^{h-1}\right)}^{T}\right)}^{-1}.$$

Here Σ_2_ is unknown since *y^h^*^−1^, *h* = *u* + 1,⋯,*k* are not available at time *t_u_*. To solve this problem, we simply ignore the variations in *W^u^*^−1^, ⋯,*W^k^*^−1^ and use *W^u^*^−1^ to replace all the other *W* s to generate an approximate distribution. Then we use Metropolis-Hastings algorithm to find the acceptance ratio and adjust samples to the right distribution. Let all *W* s equal to *W^u^*^−1^, Σ_2_ can be simplified as (*T* − *t_u_*)*σ*^2^((*I*−*αW^u^*^−1^)(*I*−*αW^u^*^−1^)^T^)^−1^, which is (*T*−*t_u_*)/(*t_u_*−*t_u_*_−1_)·Σ_1_. Therefore, we propose to sample *z^u^* according to the normal distribution with mean (*t_u_*−*t_u_*_−1_)/(*T*−*t_u_*_−1_)·*X* and variance (*T*−*t_u_*)/(*T*−*t_u_*_−1_)·Σ_1_.

### 2.9. Sample Hidden Variables Conditional on Observed Data

Remember that the observed data are *y*^0^, *z*^0^, *y*^T^, *z*^T^ and the hidden variables are *k*, *t*_1_*,*⋯*t_k_*, *y*^1^,⋯, *y^k^*^−1^, *z*^1^,⋯, *z^k^*. With known *y*^0^, the network variables *y*^1^,⋯, *y^k^*^−1^ can also be written as (*i*_(1)_, *j*_(1)_), ⋯, (*i*_(_*_k_*_−1)_, *j*_(_*_k_*_−1)_). With known *z*^0^, the behavior variables *z*^1^,⋯, *z^k^* can also be written as Δ*z*(*t*_0_, *t*_1_),⋯, Δ*z* (*t_k_*_−1,_*t_k_*). The following sampling steps will sample the above hidden variables conditional on *y*^0^, *z*^0^, *y*^T^, *z*^T^.

- Sample *k*: let *d* be the number of edges (*i*, *j*) such that $y_{i,j}^{0}\neq y_{i,j}^{T}$. Then it must follow that *k* = *d* + 2*a* for some *a* = 0,1, ⋯.
  - If *d* is even,
    $$P\left\{k=d+2a\right\}=\frac{\exp\left(-\lambda T\right){\left(\lambda T\right)}^{k}}{k!\left(\frac{1+\exp\left(-2\lambda T\right)}{2}-\sum_{u=0}^{d/2-1}\frac{\exp\left(-\lambda T\right){\left(\lambda T\right)}^{2u+2a}}{(2u+2a)!}\right)}.$$
  - If *d* is odd,
    $$P\left\{k=d+2a\right\}=\frac{\exp\left(-\lambda T\right){\left(\lambda T\right)}^{k}}{k!\left(\frac{1-\exp\left(-2\lambda T\right)}{2}-\sum_{u=0}^{(d-1)/2}\frac{\exp\left(-\lambda T\right){\left(\lambda T\right)}^{2u+2a-1}}{(2u+2a-1)!}\right)}.$$
- Sample *t*_1,_⋯_,_ *t_k_* conditional on *k*: ordered uniform (0,*T*).
- Sample (*i*_(1)_, *j*_(1)_), *z*^1^,⋯,(*i*_(_*_k_*_−1)_, *j*_(_*_k_*_−1)_), *z^k^*^−1^ conditional on others using the following procedure.
  1. Sample Δ*z* (*t*_0_,*t*_1_) from the following multinormal distribution, conditional on *y*^0^, *z*^0^, *y*^T^, *z*^T^, *k*, *t*_1,_⋯_,_ *t_k_*:
    $$N\left(\frac{t_{1}-t_{0}}{T-t_{0}}\left(z^{T}-z^{0}\right),\frac{\left(T-t_{1}\right)(t_{1}-t_{0})\sigma^{2}}{T-t_{0}}{\left(\left(I-\alpha W^{u-1}\right){\left(I-\alpha W^{u-1}\right)}^{T}\right)}^{-1}\right)$$
    and evaluate the density function of the above normal distribution at the realized value Δ*z*(*t*_0_,*t*_1_)′, which is denoted by *q*_1_(*z*′).
  2. Sample (*i*_(1)_, *j*_(1)_) = (*i*, *j*) conditional on *k* = *d* + 2*a*, *y*^0^, *z*^1^ = *z*_0_ + Δ(*t*_0_,*t*_1_):
    - Define the important list to be *L* = {(*i*_1_, *j*_1_),⋯, (*i_d_*, *j_d_*)}, where $y_{i_{u}j_{u}}^{0}\neq y_{i_{u}j_{u}}^{T}$ for *u* = 1,⋯,*d*.
    - If *a* > 0, sample and select an edge (*i*, *j*), from all *n* (*n*−1) candidates with probability exp((2*y_ij_*−1)*θ*^T^*δ_ij_*(*y*^0^, *z*^1^))/Σ*_i_*_′≠_*_j_*_′_exp((2*y_i_*_′_*_j_*_′_−1)*θ*^T^*δ_i_*_′_*_j_*_′_ (*y*^0^, *z*^1^)). Then
      - If (*i*, *j*) ∈ 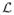, delete (*i*, *j*) from 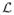, and change *d* to *d*−1.
      - If (*i*, *j*) ∉ 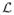, add (*i*, *j*) to 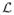, change *d* to *d* + 1, and change *a* to *a*−1.
    - If *a* = 0, sample and select (*i*, *j*) from 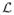 with probability exp((2*y_ij_*−1)*θ*^T^δ*_ij_*(*y*^0^, *z*^1^))/ 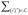
exp((2*y_i_*_′_*_j_*_′_−1)*θ*^T^*δ_i_*_′_*_j_*_′_(*y*^0^, *z*^1^)). Then delete (*i*, *j*) from 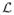, and change *d* to *d*−1.
    - Denote the probability from the situlation of *a* > 0 or *a* = 0 by *r*_1_(*y*′).
  3. Likewise, sequentially sample Δ*z* (*t*_1_,*t*_2_), (*i*_(2)_, *j*_(2)_), ⋯, Δ*z* (*t_k_*_−2_,*t_k_*_−1_), (*i*_(_*_k_*_−1)_, *j*_(_*_k_*_−1)_), and finally Δ*z* (*t_k_*_−1_, *t_k_*), and evaluate the quantities *q*_2_(*z*′),⋯,*q_k_* (*z*′) and *r*_2_ (*y*′),⋯, *r_k_*_−1_(*y*′).
  4. Use the Metropolis Hastings algorithm to decide whether to accept the generated sample (*y*′, *z*′) or not.
    The acceptance ratio is
    $$\min\left\{1,\frac{L(y^{\prime })q_{1}(z)\cdots q_{k}(z)r_{1}(y)\cdots r_{k-1}(y)}{L(y)q_{1}(z^{\prime })\cdots q_{k}(z^{\prime })r_{1}(y^{\prime })\cdots r_{k-1}(y^{\prime })}\right\}$$
    where *L*( ) is the complete data likelihood function.

## 3. Results

We used the Add Health “saturation sample” data to check the reasonableness of our assumptions and to perform simulation studies. First, we show results based on the ABSM model; next we compare these results with our co-evolution model.

The Add Health saturation sample data are based on adolescents in 16 high schools where all students in a given school were asked to participate. There are two waves (1 year apart) of friendship network data, including environmental variables and self-reported height/weight. We focus on one school called “Jefferson High” as in [16] [17], where over 99% students are white. In this data set, the sample size with complete data over two waves is 624, among which 52.7% are males. The grade levels range from 9 to 11, the average BMI is 23.1 with SD being 4.4 and the average outdegree (number of friends named) of the network is 4.0 with SD being 2.1.

### 3.1. Results for ABSM Models

The results based on ABSM are in Table 1. The parameter of waiting time for the opportunity of change is *λ*_total_ = 624 × 12.29+4.17 ) = 10,271. That is, the average waiting time between two adjacent opportunities of change is 1/10271×36524 = 0.85 (hour). The overall mean of BMI is 23.10 and the average similarity score is 0.8619. The average sex similarity score is 0.5005 and grade similarity is 0.6598.

The estimated network objective function is

$$f_{i}(y,z)=-3.4228\sum_{j}y_{ij}+2.3341\sum_{j}y_{ij}y_{ji}+0.4957\sum_{j}y_{ij}\sum_{h}y_{ih}y_{hj}+0.058\sum_{j}y_{ij}I\left\{s_{i}=s_{j}\right\}+0.5417\sum_{j}y_{ij}I\left\{g_{i}=g_{j}\right\}+0.3901\sum_{j}y_{ij}\left(1-|z_{i}-z_{j}|/32-0.8619\right).$$

where *s* represents sex, *g* grade, and *z* BMI. The estimated behavior objective function is

$$g_{i}\left(y,z\right)=0.1571\left(z_{i}-23.098\right)+0.0144{\left(z_{i}-23.098\right)}^{2}\;13.9074\sum_{j}y_{ij}\left(1-\left|z_{i}-z_{j}\right|/32-0.8619\right)/\sum_{j}y_{ij}.$$

For example, consider the behavior evolution for individuals who have no friends. The estimated behavior objective function becomes

$$g_{i}\left(y,z\right)=0.1571\left(z_{i}-23\right)+0.0144{\left(z_{i}-23\right)}^{2}=0.0144{\left(z_{i}-17.5\right)}^{2}+\text{constant}.$$

The probabilities for BMI evolution are shown in Table 2. The results indicate that for individuals whose BMI is greater than 17.5 there is a higher probability of an increase in BMI, which is consistent with the observed propensity for BMI to “track” over time [18] [19]. However, for individuals whose BMI is less than 17.5, the results indicate a higher probability of decrease in BMI; this may not be reasonable.

### 3.2. Validation of Assumptions in the Joint Evolution Model

**Figure 1:**
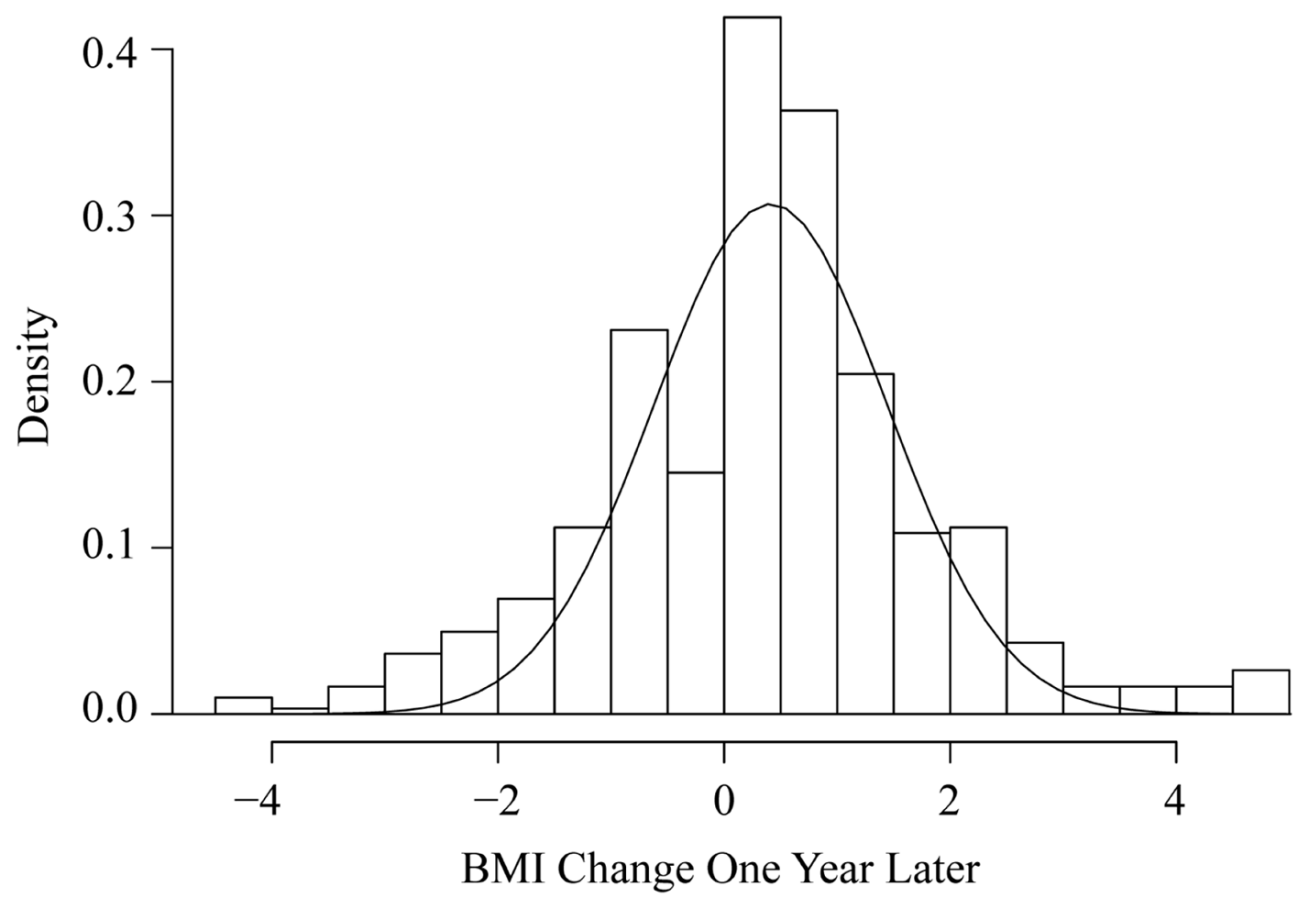
The histogram of BMI change for the school of Jefferson High in Add Health data.

Using the Add Health data for the school of Jefferson High, we can draw the histogram of BMI change and screen time change between these two waves (Figure 1). From Figure 1, we see that the normality assumption is not perfectly satisfied due to larger amount of observations around zero. However, the distributions are approximately symmetric, which is usually sufficient in a linear model if sample size is moderately large (for example, greater than 30).

**Figure 2:**
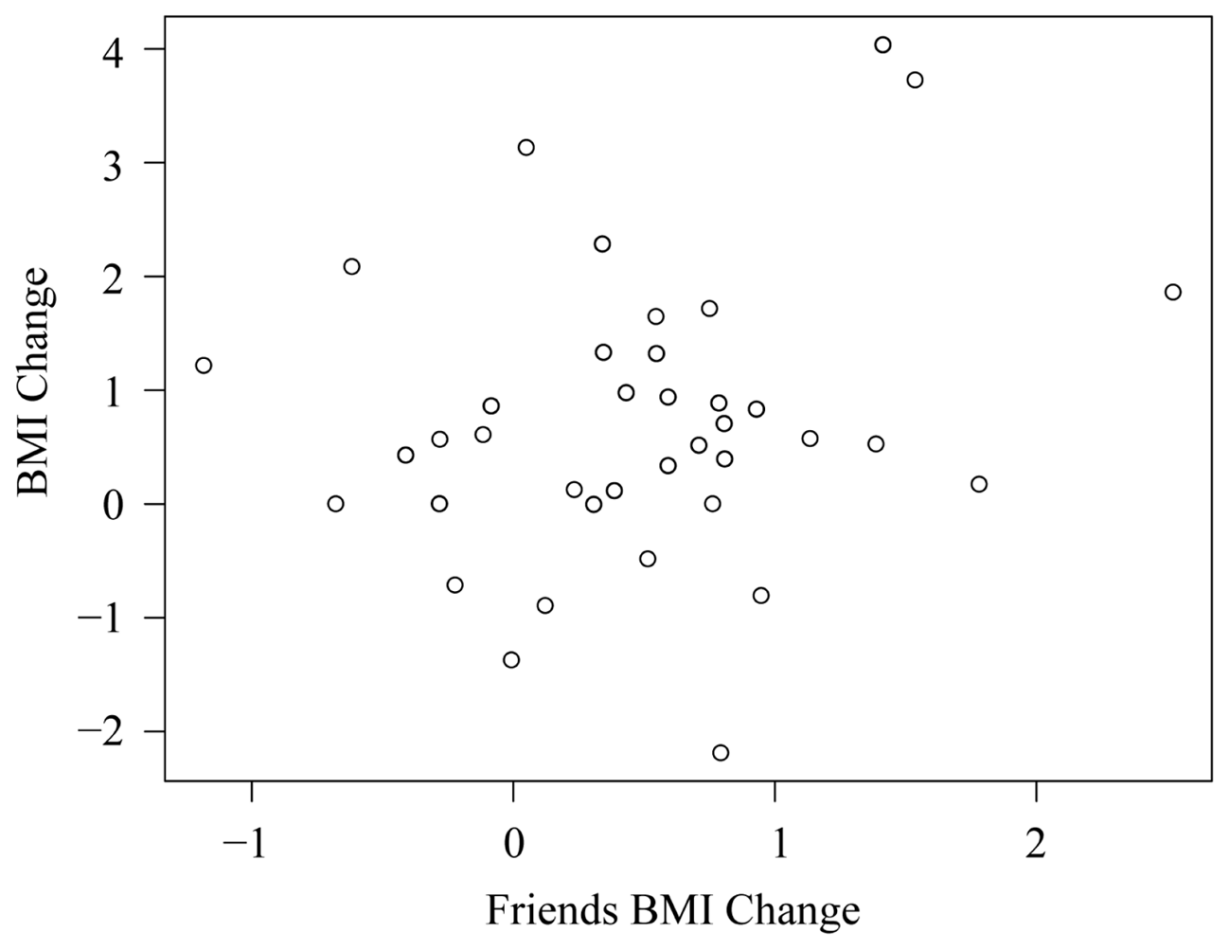
The scatter plot of individuals’ BMI change versus average friends’ BMI change for the school of Jefferson High in Add Health data.

We also draw the scatter plot of individual’s BMI change versus average friends’ BMI change to check linearity assumption. The plot in Figure 2 suggest weak linear relationship between these two variables. Note that to draw this plot, we consider only friends who were nominated at both waves so that the BMI change comparison is valid. Therefore, the relationship shown reflect only part of the data, which contributes to the weakened linear relationship. These findings suggest that our assumptions are approximately satisfied.

### 3.3. Simulation Study

To simulate a realistic network with reasonable BMI values assigned to each individual, we randomly sampled 30 individuals (from the same school) in the Add Health data. The average BMI of selected individuals is 22.9 kg/m^2^. We then create an initial network using Bernulli graph with density = 0.3.

We specified that network and BMI would evolve for 60 days using the following parameters values: *λ* = 1, *γ* = 0.001, *α* = 0.1, *σ* = 0.1, and *θ* = (−3.42,2.33,0.50,0.39) with corresponding statistics of outdegree, reciprocity, transitive triplets and BMI total similarity. In the simulated data set, the number of network change events = 65, within which 21 edges change from 1 to 0 and 44 change from 0 to 1. The average BMI after 60 days is 23.6 and network density 0.33.

Apply the EM procedure described in Methods section, we obtained the following parameter estimations (Table 3). From the table, we see that some of the parameter estimates, such as event rate *λ*, network effect *α*, and coefficient of out degree *θ*_1_ are relatively accurate. The variance *σ*^2^ is underestimated. The other parameters are not significant comparing to zero. This suggest that our algorithm can find reliable parameter estimates for those that are significantly different from zero.

The explanation of the above parameters are mostly straight forward. For example, the rate of events *λ* = 1.08 indicates that on average, there is 1.08 edge change during one unit time (one day in this example). The coefficient of trends *γ* is not distinguishable from zero, which means that there is no significant trend of BMI increase or decrease during these 60 days. The parameter that is of most interest is the network effect *α*, which is 0.12 in this example. This means that whenever an individual’s friends’ average BMI increases/decreases by one unit, this individual’s BMI is expected to increase/decrease by 0.12.

### 3.4. Application to Real Data

Since our model cannot deal with a network as large as 624 individuals, we include only students in grade 11 in this application. The sample size here is 110. We first run the ABSM model using RSiena (Table 4). Then we fit in our joint co-evolution model (Table 5).

Compare results from Table 4 and Table 5. We found out that the results for network evolution are similar from both models. This is because we are using the same network evolution models. The different behavior models have only limited effect on the network evolution process. Both model suggest that there is no effect of selection or influence. That is, similarity in BMI does not affect the process of making friends; an individual’s friends’ BMI change does not affect his/her own BMI. Note that when we use the complete data of 624 individuals, we got significant effect of selection (*p* = 0.0309) and influence (*p* = 0.0002). The insignificant results here are due to reduced sample size and lost information in missing values.

## 4. Discussion

We have developed a joint social network and behavior evolution model. In our model, behavior changes are consistent over time. That is, Δ*Z* and Δ*Z*_1_ + Δ*Z*_2_ have the same distributions. Our model is robust to scaling of behavior variables, and parameter values are easy to interpret. In addition, this framework may be readily expanded to study valued networks.

The field of social network analysis is a relatively young field. However, useful contributions are being made today. The range of applications is vast, from the contagion of health behaviors described in this paper [20], to the study of group formations in human societies [21]. Further advances will require improved statistical methods (to deal with different types of behaviors departing from the discrete choice model), as well as more extensive empirical data sets incorporating social networks. Many future studies will use continuous outcome measures; we hope the method presented here will be valuable in extending the ABSM to such outcomes.

Our model does require intensive computation. However, we are confident that more efficient algorithms can be developed. Though our model requires specific assumptions, we have demonstrated that these assumptions are reasonably easy to satisfy using real data. Sensitivity analysis will ultimately be required to determine if our model works well when some of the assumptions are violated.

## Figures and Tables

**Table 1:** Estimated ABSM for the school of Jefferson High.

	Function	Estimate	S.E.	P value
Network dynamics				
1. Rate: rate friendship		12.2900	(0.4620)	<1e–4
2. Eval: outdegree (density)	Σ*_j_y_ij_*	−3.4228	(0.0370)	<1e–4
3. Eval: reciprocity	Σ*_j_y_ij_y_ji_*	2.3341	(0.0664)	<1e–4
4. Eval: transitive triplets	Σ*_j_y_ij_*Σ*_h_y_ih_y_hj_*	0.4957	(0.0268)	<1e–4
5. Eval: same sex	Σ*_j_y_ij_I*{*s_i_* = *s_j_*}	0.0580	(0.0451)	0.1984
6. Eval: same grade	Σ*_j_y_ij_I*{*g_i_* = *g_j_*}	0.5417	(0.0501)	<1e–4
7. Eval: BMI similarity	Σ*_j_y_ij_*(0.1381−|*z_i_* − *z_j_*|32)	0.3901	(0.1807)	0.0309

Behavior dynamics				
8. Rate: rate BMI period 1		4.1708	(0.3577)	<1e–4
9. Eval: BMI linear shape	(*z_i_* − 23.098)	0.1571	(0.0275)	<1e–4
10. Eval: BMI quadratic shape	(*z_i_* − 23.098)^2^	0.0144	(0.0066)	0.0291
11. Eval: BMI similarity	Σ*_j_y_ij_*(0.1381−|*z_i_* − *z_j_*|32)	13.9074	(3.7561)	0.0002

**Table 2:** BMI evolution probabilities for individuals with no friends.

BMI	−1	Same	+1
14	0.000	0.522	0.478
15	0.359	0.330	0.311
17	0.340	0.330	0.330
18	0.330	0.330	0.340
30	0.224	0.316	0.460
45	0.124	0.270	0.605
46	0.309	0.691	0.000

**Table 3:** MLE parameter estimations using simulated data.

Description	Parameter	True value	MLE (S.E.)
Rate of events	*λ*	1	**1.08 (0.17)**
Coeff. of trend	*γ*	0.001	0.002 (0.004)
Network effect	*α*	0.1	**0.12 (0.03)**
Standard deviation of noise	*σ*	0.1	**0.07 (0.02)**
Coeff. of outdegree	*θ*_1_	−3.43	−**3.72 (0.35)**
Coeff. of reciprocity	*θ*_2_	2.33	1.63 (1.22)
Coeff. of transitive triplets	*θ*_3_	0.50	0.39 (0.79)
Coeff. of BMI similarity	*θ*_4_	0.39	0.71 (1.26)

**Table 4:** Estimated ABSM using Jefferson High grade 11 data.

	Estimate	S.E.	P value
Network dynamics			
1. Rate: rate friendship	4.7385	(0.4698)	<1e–4
2. Eval: outdegree (density)	−3.0952	(0.1425)	<1e–4
3. Eval: reciprocity	2.3959	(0.2244)	<1e–4
4. Eval: transitive triplets	0.5471	(0.0800)	<1e–4
5. Eval: same sex	0.2099	(0.1517)	0.083
6. Eval: BMI similarity	0.5425	(0.6607)	0.206

Behavior dynamics			
7. Rate: rate BMI period 1	2.8444	(0.5431)	<1e–4
8. Eval: BMI linear shape	0.1737	(0.0853)	0.021
9. Eval: BMI quadratic shape	−0.0504	(0.0225)	0.013
10. Eval: BMI similarity	1.6567	(1.8186)	0.181

**Table 5:** MLE parameter estimations using Jefferson High grade 11 data.

Description	Parameter	MLE (S.E.)
Rate of events	*λ*	2.59 (1.22)
Coeff. of trend	*γ*	0.00 (0.00)
Network effect	*α*	0.02 (0.05)
Standard deviation of noise	*σ*	1.20 (0.39)
Coeff. of outdegree	*θ*_1_	3.25 (0.15)
Coeff. of reciprocity	*θ*_2_	2.19 (0.38)
Coeff. of transitive triplets	*θ*_3_	0.59 (0.07)
Coeff. of BMI similarity	*θ*_4_	0.36 (0.41)
